# Assessing Nonacceptance of the Facial Appearance in Adult Patients After Complete Treatment of Their Rare Facial Cleft

**DOI:** 10.1007/s00266-012-9897-y

**Published:** 2012-04-13

**Authors:** Marijke E. P. van den Elzen, Sarah L. Versnel, Hugo J. Duivenvoorden, Irene M. J. Mathijssen

**Affiliations:** 1 Department of Plastic and Reconstructive Surgery, Erasmus University Medical Centre, Room Ee 15.91, Dr. Molewaterplein 50, 3015GE Rotterdam, The Netherlands; 2 Department of Medical Psychology and Psychotherapy, NIHES, Erasmus University Medical Centre, Rotterdam, The Netherlands

**Keywords:** Acceptance, Adults, Congenital, Facial disfigurement, Questionnaire, Satisfaction

## Abstract

**Background:**

Treatment of patients with severe congenital facial disfigurements is aimed at restoring an aesthetic and functional balance. Besides an adequate level of satisfaction, an individual’s acceptance of facial appearance is important to achieve because nonacceptance is thought to lead to daily psychological struggles. This study objectified the prevalence of nonacceptance among adult patients treated for their severe facial clefts, evaluated risk factors, and developed a screening tool.

**Methods:**

The study included 59 adults with completed treatment for their severe facial cleft. All the patients underwent a semistructured in-depth interview and filled out the Body Cathexis Scale.

**Results:**

Nonacceptance of facial appearance was experienced by 44 % of the patients. Of the nonaccepting patients, 72 % experienced difficulties in everyday activities related to their appearance versus 35 % of the accepting patients. Acceptance did not correlate with objective severity or bullying in the past. Risk factors for nonacceptance were high self-perceived visibility, a troublesome puberty period, and an emotion-focused coping strategy. Also, the presence of functional problems was shown to be highly associated.

**Conclusions:**

The objective severity of the residual deformity did not correlate with the patients’ acceptance of their facial appearance, but the self-perceived visibility did correlate. The process of nonacceptance resembles the process seen in patients with body dysmorphic disorders. Surgical treatment is no guarantee for an improvement in acceptance and is therefore discouraged for patients who match the risk factors for nonacceptance unless it solves a functional problem. The authors therefore recommend screening patients for nonacceptance and considering psychological treatment before surgery is performed.

**Level of Evidence III:**

This journal requires that authors assign a level of evidence to each article. For a full description of these Evidence-Based Medicine ratings, please refer to the Table of Contents or the online Instructions to Authors at www.springer.com/00266.

Patients with severe facial clefts experience multiple operations from a very young age until adulthood. Treatment is aimed at restoring an aesthetic and functional balance. Hopefully, this will lead to a satisfied and self-accepting patient in the long term, so a “normal life” can be lived. It must be stated that satisfaction and acceptance are not the same: A patient may be dissatisfied with the end result but accept his or her residual deformity.

The abundant number of studies on acceptance covers cohorts of patients with a specific chronic disease or chronic pain [4–6, 9, 12, 13, 15, 16, 18, 20, 21, 26, 27]. However, reports specifically on acceptance of appearance are scarce [8, 20]. Within the published studies, acceptance is defined as a willingness to have unwanted experiences on some occasions, with reorientation toward positive everyday activities and functioning [16]. Studies concerning patients with chronic diseases or chronic pain have shown that nonacceptance leads to psychological distress and disability, reduced subjective health, depression, anxiety and emotional instability, and avoidance [5, 6, 9, 14, 16, 18, 20, 21, 26, 27].

Earlier studies investigating patients with severe congenital facial disfigurement reported that the main problems are on the social functioning level due to prejudices and reactions of disapproval by others [17, 25]. This results in a fear-avoidance behavior, with patients avoiding confrontations so they will not experience stress [11, 17]. The model of avoidance behavior is based on a model of exaggerated pain perception of patients with chronic pain who avoid movements and situations so they will not experience pain. Because the reaction of avoidance in patients with chronic pain and facial disfigurement is similar, perhaps the principals of acceptance also may be alike. In view of the fact that amelioration of acceptance in patients with chronic diseases or pain may induce an improved level of psychological well-being, less psychological distress, and a higher level of emotional stability [5, 6, 9, 14, 16, 18, 20, 21, 26, 27], this also might be applicable for patients with congenital severe facial disfigurements.

In our opinion, evaluating the satisfaction that patients with severe congenital disfigurement have about their appearance is not enough. A patient’s acceptance of his or her facial appearance is of similar clinical importance. Recognizing a patient at risk for nonacceptance is crucial for offering the best treatment to ameliorate acceptance and possibly thereby to enhance psychosocial functioning.

Our first objective was to investigate the prevalence of patients with nonacceptance and to identify risk factors for the development of this nonacceptance. Because most studies investigate the entire group of patients, it can be hard to identify an individual patient. Therefore, the second objective was to construct a short and specific screening tool tailored to test for nonacceptance of an individual patient.

## Material and Methods

### Study Population

Only adult patients with a severe congenital facial deformity were recruited. Of the 123 selected patients with a rare facial cleft (e.g., midline and oblique facial cleft, Treacher Collins syndrome) who had undergone surgery between 1969 and 2009 at the Department of Plastic and Reconstructive Surgery of the Erasmus University Medical Center or Sophia Children‘s Hospital, Rotterdam, the Netherlands, only 75 were invited to participate in this study. This patient cohort was chosen because they encompassed deformities in all facial units in different sequences [24, 29]. We chose to omit hemifacial microsomia because it represents a relatively large subpopulation and thus would overrepresent a specific type of deformity.

A total of 48 patients were excluded from the study because they met one or more of the following exclusion criteria: deceased (*n* = 4), incomplete data (*n* = 9), age younger than 18 years (*n* = 32), mentally handicapped (*n* = 1), blind (*n* = 1), and insufficient command of the Dutch language (*n* = 1).

### Design and Procedure

A clinical-empirical cross-sectional study was designed and conducted. Ethical approval was received from the board of the Medical Ethical Committee of the Erasmus University Medical Centre Rotterdam (MEC-2006-121).

By mail, patients were sent a cover letter, a patient information form, a questionnaire, and an informed consent form to sign. After the completed questionnaire was returned, an appointment was made for the interview, which was held at the patient’s home address.

### Questionnaire

#### Body Cathexis Scale

A prior study introduced the modified version of the Body Cathexis Scale (BCS): the Facial BCS. Both the original version [23] and the Facial BCS were used in the current study. The original BCS contains 46 items with a 5-point response scale to measure the function of the body parts and the patient’s satisfaction with this function.

The original BCS comprises the whole body, including the face as well, but it does not comprise all the important facial parts and functions. Therefore, in the Facial BCS, extra facial parts and functions were added. A total of five scores were calculated: the original BCS, the Facial BCS, and three subscores (the BCS appearance-of-face, the BCS function-of-face, and the BCS whole-body-without-face. All the scores showed good internal consistency reliability [30]. A validated Dutch version of the original BCS is available [3].

### Interview

The semistructured in-depth interview covered the potential predictive factors chosen and divided into external factors (upbringing, religion, and bullying) and internal factors (coping styles, value of the opinion of others, troublesome puberty, troubles in everyday activities, self-perceived visibility, and whether the patient had the desire to undergo psychological treatment). This methodology was chosen to collect data in a qualitative manner because standardized scales might be insensitive to the particular issues of these patients [25].

All the interviews were conducted by a single researcher (SLV). The majority of the questions were open-ended, and responses were followed by a question elaborating on the motives behind the patient’s answer. The interview data were assessed using a thematic analysis on the basis of which themes in the qualitative material could be identified by a coding scheme.

### Potential Predictive Factors

#### Objective Severity of Facial Disfigurement

Besides the patients’ answers in the interview conducted to cover the external and internal potential predictive factors, the severity of the residual facial disfigurement of each patient was independently scored by two experts using the scoring list according to Versnel et al. [31] for facial disfigurement. Recent postoperative standardized photographs of all the patients were used. The average score was calculated in case of different scores.

### Measurement of Nonacceptance of Facial Appearance

The presence of nonacceptance was not queried as a direct question to the patient. It was calculated by answers on multiple questions derived from the interview. The questions concerning nonacceptance were composed by two of the authors In this study, patients were scored as nonaccepting if they encountered true difficulties by looking in a mirror or if they reported not being used to their facial appearance or frequently having psychological struggles due to their appearance with a seriously severe character. The questions in this measurement were chosen because they represent general everyday pursuits unthreatening to answer but very relevant for acceptance. The questions are not about whether the patients like their appearance or not about how much negative impact these unwanted experiences gave them and thus indirectly the willingness to experience them.

### Statistical Analyses

As a measure of central tendency for continuous data, we used mean ± standard deviation as a measure of dispersion. In case of categorical data, the percentages were calculated. Furthermore, the method of logistic regression analysis was used, with nonacceptance coded as 1 and acceptance as coded as 0. As a measure of individual performance of the predictor variable, the odds ratio (OR) was estimated, including the corresponding 95% confidence interval (95% CI). All the analyses were adjusted for gender and age. The level of statistical significance was fixed at 0.05 (two-tailed). For statistical analysis, we used the Statistical Package for the Social Sciences (SPSS) for Windows, version 15 (SPSS, Chicago, IL, USA).

## Results

### General Characteristics

Of the 75 rare facial cleft patients who met our inclusion criteria, 59 (79 %) participated in the study. The remaining 16 patients refused for the following reasons: did not respond (*n* = 8, 4 lived abroad), found treatment too traumatic (*n* = 3), had interviews with the media about their disfigurement and did not want to talk anymore (*n* = 2), and had emotional difficulties (*n* = 3). The patient characteristics are presented in Tables 1 and 2.

**Table 1:** Patient characteristics

	*n* = 59
Gender (%)	
Male	32.2
Female	67.8
Age (years)	
Mean	34.05
SD	12.92
Min–Max	18–74
Education level (%)	
Primary school^a^	35.1
High school^a^	47.4
Postgraduation^a^	17.5
Severity facial deformity	
Mean score	13.90
SD	7.65

*SD* standard deviation, *Min–Max* minimum–maximum

^a^Represents column percentages

**Table 2:** Details on patient characteristics

Patient no.	Type of clefts^a^	Uni- or bilateral	Total no. of surgeries	OSRFD	Gender
1	2, 3, 4, 5, 10	Bilateral	16	19	Female
2	Pure midline (0–14)		14	4	Female
3	Treacher-Collins (6, 7, & 8)		1	26	Male
4	Treacher-Collins (6, 7, & 8)		1	5	Female
5	2, 3, 11	Unilateral	18	18	Male
6	CFND (0–14 + craniosynostose)		4	4	Female
7	Pure midline (0–14)		4	7	Female
8	Treacher-Collins (6, 7, & 8)		3	8	Female
9	Treacher-Collins (6, 7, & 8)		7	19	Male
10	Pure midline (0–14)		6	10	Female
11	0, 2, 3, 4, 5, 9, 11	Bilateral	9	26	Female
12	ALX3 (0–14)		5	10	Female
13	4	Bilateral	10	20	Male
14	Treacher-Collins (6, 7, & 8)		1	1	Female
15	1, 2, 3	Unilateral	26	23	Male
16	CFND (0–14 + craniosynostose)		8	20	Female
17	Treacher-Collins (6, 7, & 8)		2	14	Male
18	2, 3, 7, 8, 11	Bilateral	7	14	Female
19	0, 1, 2, 3, 10	Bilateral	14	23	Female
20	Treacher-Collins (6, 7, & 8)		6	4	Male
21	0, 2, 3	Unilateral	3	23	Female
22	Treacher-Collins (6, 7, & 8)		1	13	Male
23	CFND (0–14 + craniosynostose)		7	11	Female
24	Treacher-Collins (6, 7, & 8)		4	5	Female
25	3	Bilateral	10	14	Male
26	Pure midline (0–14)		9	7	Female
27	0, 2, 3	Bilateral	2	6	Female
28	3	Unilateral	4	12	Female
29	Treacher-Collins (6, 7, & 8)		3	10	Female
30	2, 3	Unilateral	12	9	Male
31	Treacher-Collins (6, 7, & 8)		3	11	Female
32	Treacher-Collins (6, 7, & 8)		5	6	Female
33	2, 3	Unilateral	11	6	Female
34	1, 2, 3	Unilateral	15	7	Female
35	3, 4	Unilateral	10	16	Female
36	CFND (0–14 + craniosynostose)		10	19	Male
37	3, 4	Unilateral	5	12	Female
38	Treacher-Collins (6, 7, & 8)		5	10	Female
39	CFND (0–14 + craniosynostose)		2	11	Female
40	CFND (0–14 + craniosynostose)		2	10	Female
41	ALX3 (0–14)		15	6	Female
42	Treacher-Collins (6, 7, & 8)		3	4	Female
43	2, 3	Unilateral	18	20	Male
44	Treacher-Collins (6, 7, & 8)		1	16	Female
45	0, 3	Bilateral	3	4	Male
46	0, 2, 3, 4, 11	Bilateral	12	10	Male
47	3	Unilateral	15	22	Female
48	ALX3 (0–14)		15	21	Male
49	Treacher-Collins (6, 7, & 8)		6	17	Female
50	CFND (0–14 + craniosynostose)		2	9	Female
51	2, 3	Unilateral	16	8	Female
52	Treacher-Collins (6, 7, & 8)		7	20	Male
53	Treacher-Collins (6, 7, & 8)		2	14	Male
54	0, 2	Unilateral	5	2	Female
55	Treacher-Collins (6, 7, & 8)		5	11	Female
56	1, 2, 3, 4	Unilateral	11	13	Female
57	0, 1, 2	Unilateral	9	15	Female
58	Treacher-Collins (6, 7, & 8)		3	15	Male
59	1, 2, 3, 11	Unilateral	14	22	Male

*OSRFD* Objective Severity of Residual Facial Deformity according to the Versnel scoring list [31]; CFND; ALX

^a^Some patients had multiple clefts simultaneously

### Prevalence of Nonacceptance

This study first aimed to objectify the proportion of patients experiencing nonacceptance with their facial appearance (44 % of all the patients). Of the patients experiencing nonacceptance, 72 % reported troubles in everyday activities due to their appearance versus 35 % of accepting patients, which is a significant difference (*p* = 0.01). Also, the patients’ desire to undergo psychological treatment was significantly different between the nonaccepting (48 %) and accepting (11 %) patients (*p* = 0.002).

### Predictive Factors

The risk factors associated with nonacceptance are presented in Table 3. Because gender was disproportionally represented in this population and age had a significant correlation with nonacceptance (*p* = 0.04), all outcomes were corrected for both age and gender. Educational level was not associated with acceptance and therefore was omitted.

**Table 3:** Association of nonacceptance with potential predictive factors

Risk factors	OR	95% CI		*p*-value
External factors^a^				
Objective severity	1.12	0.99	1.27	0.09
Religious propensity	1.09	0.34	3.48	0.89
Protective upbringing	0.34	0.10	1.15	0.08
Bullying in past	0.91	0.19	4.29	0.91
Internal factors^a^				
Avoidance coping style	0.67	0.38	1.19	0.17
Emotional coping style	3.45	1.39	8.54	**0.01**
Valuing opinion of others	1.92	0.98	3.77	0.06
Troublesome puberty	2.40	1.43	4.03	**0.00**
Self-perceived visibility	1.97	1.06	3.69	**0.03**

*OR* odds ratio, *CI* confidence interval

1 = nonacceptance; 0 = acceptance

^a^All corrected for gender and age

Bold value indicates α = <0.05

Acceptance was not associated with the external factors such as objective severity of the residual deformity, religious propensity, protective upbringing, and bullying in the past. However, the associated risk factors for nonacceptance were the internal factors of emotional coping strategy, troublesome puberty due to facial appearance, and high self-perceived visibility of the residual deformity. It must be stressed that the external factor of protective upbringing and the internal factors of valuing the opinion of others and an avoidance coping style all had a high OR but an insufficient effect to be significantly different between acceptors and nonacceptors.

### Association Between Nonacceptance and Satisfaction with Facial Appearance

Because the BCS is seen as a measurement of satisfaction, the association of the BCS and its subscales with nonacceptance was calculated, as can be seen in Table 4. Nonacceptance was highly associated with all the BCS scores (*p* ≤ 0.01) except the BCS body-without-face score. In addition, the BCS function-of-face was shown to have a remarkably high odds ratio as well (OR, 0.11).

**Table 4:** Association of Body Cathexis Scale (BCS) with nonacceptance

Scale or subscale	OR	95% CI		*p*-value
Original BCS	0.91	0.85	0.96	**0.002**
Facial BCS	0.88	0.82	0.95	**0.001**
BCS appearance-of-face	0.80	0.69	0.91	**0.001**
BCS function-of-face	0.11	0.02	0.55	**0.007**
BCS whole-body-without-face	0.90	0.77	1.06	0.20

All corrected for gender and age

*OR* odds ratio, *CI* confidence interval

1 = nonacceptance; 0 = acceptance

Bold value indicates α = <0.05

## Discussion

It must be stated that in most cases, even after optimal surgical treatment, total normalization of the facial features is seldom achieved, and a patient must face a degree of residue [28, 32]. An earlier study conducted within the same patient population learned that the vast majority of these patients (83.1 %) were not satisfied with the end result, even when an optimal reconstruction was achieved [30]. At that point, surgical options for improvement were limited. Therefore, acceptance of their own face was important to achieve, especially for the patients dissatisfied with the appearance their face.

The different numbers of patients dissatisfied with facial appearance (83 %) and those unable to accept it (44 %) clearly illustrate that these are two separate entities for outcome measurement. All the patients who could not accept the appearance of their face also were dissatisfied, whereas only 53 % of the dissatisfied patients could not accept their appearance. The patients with nonacceptance experience this on a daily basis and indicate a greater wish for psychological support.

In this study, the internal predictive factors of high self-perceived visibility of the residual deformity, psychological troubles during puberty, and an emotional coping style were associated with nonacceptance. However, not all the potential predictive factors showed a significant difference between groups of accepting and nonaccepting patients, perhaps due to the relatively small group of patients enrolled in this study. However, looking at the high OR and the clear significant tendency, it is most likely that if our study population had been larger, the factors of protective upbringing, valuing the opinion of others, and an avoidance coping style also would have been differentiating factors between acceptors and nonacceptors. Moreover, the relatively small group also limited the number of risk factors that could be investigated. In addition, the retrospective nature of some questions in the interview might have induced a bias. However, on the other hand, this is how the patient experienced the event in hindsight.

Ideally, a patient at risk for nonacceptance should be identified within a few minutes at the outpatient clinic. Most of the published studies concerning acceptance of appearance are not appropriate for an outpatient clinic setting, particularly due to their length. Finding an individual patient at risk can therefore be difficult.

**Fig. 1:**
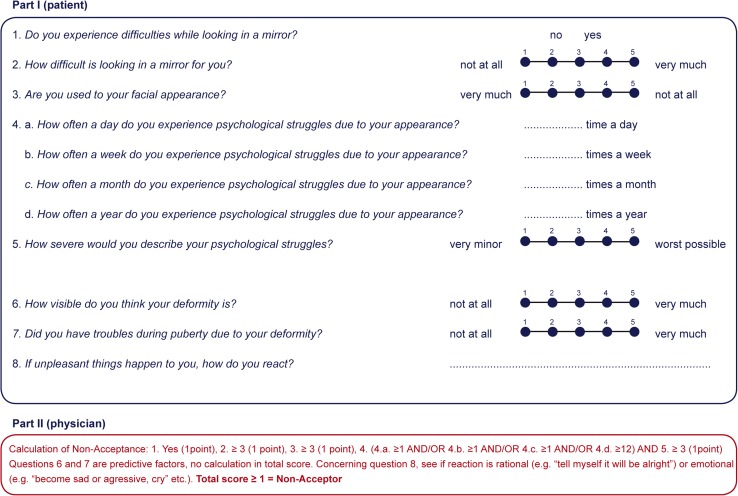
Questionnaire for nonacceptance

To tackle this problem, we constructed a screening tool for nonacceptance (Fig. 1) according to questions and predictive factors derived from the interview used in this study. For the reason that this study is only descriptive and explorative toward the screening tool for nonacceptance, further research is necessary to validate and support our screening tool. At this writing, the tool is being tested at the outpatient clinic of the craniofacial team. In addition, this screening tool and the prevalence of nonacceptance must be tested with different types of patients (e.g., reconstructive and aesthetic patients) before the conclusions reached in this study can be extrapolated to other patient groups.

Our results on nonacceptance and its predictive factors imply that amelioration of acceptance with the deformed facial appearance in these patients can be achieved by adjustment to these internal processes and most likely by professional psychological help. The high ratio of patients with a desire for psychological treatment (48 %) also reflects this. Studies on acceptance of chronic pain have shown promising results with cognitive behavioral therapy [10, 33]. Because both the patients with chronic pain and our facially disfigured patients have comparable patterns of fear avoidance and areas of psychological struggles due to their ailment [11, 17], the results of psychological treatment might be extrapolated to patients with severe congenital facial disfigurement. In addition, the importance of the upbringing and the troubles experienced during puberty illustrate that acceptance may be established at a young age. Therefore, parents should know about the effect of a protective upbringing and about the standards and values they teach their children. A combined therapy of patients and their parents could therefore be helpful.

Our observation that the objective severity has no association with acceptance suggests that surgery alone might not be the answer to the problems encountered by these patients. However, surgical options to correct residual abnormalities in their faces often are available. Therefore, the question is when to operate on a nonaccepting patient. The answer to this may be found in a different group of patients. The nonaccepting patients in this study were similar in some ways to patients with body dysmorphic disorder (BDD). In short, the definition of BDD is a preoccupation with an imagined or slight physical abnormality that causes significant distress or impairment in social, occupational, or other areas of functioning [2, 22]. Nonaccepting patients with a residual deformity after completed surgical treatment of their facial cleft have a preoccupation with their deformity, which also leads to social impairment irrespective of the deformity’s severity of objective visibility.

In studies of patients with BDD, surgery rarely improves the situation [1, 7, 19]. In contrast, psychological treatment has proved to be more effective in most cases [1]. Surgical treatment for nonaccepting patients with a residual deformity after complete treatment of their facial disfigurement should therefore be reconsidered carefully because their expectations may be unrealistic.

An exception to this recommendation is a surgical procedure to solve functional problems. This study showed that a low score on the BCS function-of-face has a high association with nonacceptance. This implies that the better the function of the face, the more likely will be acceptance of the face. Therefore, a distinction should be made based on the character of the patients’ desire for additional surgery. The final recommendation therefore is to withhold surgical interventions for nonaccepting patients with a residual deformity after completed surgical treatment unless the treatment aims at restoring a functional problem.

We conclude that acceptance of one’s facial appearance is a different outcome measurement than satisfaction with one’s facial appearance and that this difference has high relevance to surgical decision making for the surgeon and also has a serious impact on social functioning for the patient.

Almost half of the adult patients with a rare facial cleft did not accept their facial appearance after completion of surgical treatment. The short questionnaire provided in this study facilitates recognition of these nonacceptors. The objective severity was not correlated with patients’ acceptance of their facial appearance, but the self-perceived visibility was correlated with their acceptance. Therefore, it is very unlikely that an additional surgical correction will change the way patients see themselves. Moreover, residual deformities will be visible even after excellent surgical results are achieved. We therefore highlight the option of not operating on these patients who after completing surgical treatment face a residual deformity unless surgery solves a functional problem.

### Extrapolation to Other Groups of Patients

As mentioned earlier, because this study covers a very specific and rare group of patients with severe facial deformities, an extrapolation of these conclusions to other groups of patients cannot be made immediately. The number of patients who experience nonacceptance (44 %) is rather large in this group. To rule out reasons other than the fact that the nonacceptance of these patients just is relatively high, we emphasize that we cannot ascribe this result to a selection bias because all the patients who met our inclusion criteria participated in this study. The 16 patients who did not respond to our invitation to participate in this study were even less courageous, emotionally struggling patients. If they had participated, it is very likely that the number of nonacceptors would have been even higher. Nevertheless, the total number of patients participating in this study was relatively small. Due to the rarity of the facial deformities studied, a larger number was not possible. However, this may have distorted the outcome of this study both by the relatively small number of patients and by the very specific group of patients. Also, this observation is made from a single measurement. To determine whether the process of nonacceptance might be dynamic, a longitudinal study would be illustrative.

In conclusion, to validate the described screening tool and to estimate the prevalence of nonacceptance among other types of patient groups, this study must be conducted with other different types of surgical subgroups such as reconstructive and aesthetic patients and with a larger number of patients before the conclusions reached in this study can be extrapolated to other patient groups.
